# Increased Viral Dissemination in the Brain and Lethality in MCMV-Infected, Dicer-Deficient Neonates

**DOI:** 10.3390/v7052308

**Published:** 2015-05-06

**Authors:** Eleonore Ostermann, Cécile Macquin, Wojciech Krezel, Seiamak Bahram, Philippe Georgel

**Affiliations:** 1 Immunorhumatologie Moléculaire, INSERM UMR S_1109, Centre de Recherche en Immunologie et Hématologie, Faculté de Médecine, Fédération de Médecine Translationnelle de Strasbourg (FMTS), Université de Strasbourg, Strasbourg. 1, Place de l’Hôpital, 67085 Strasbourg Cedex, France; E-Mails: eleonore.ostermann@hpi.uni-hamburg.de (E.O.); macquin@unistra.fr (C.M.); siamak@unistra.fr (S.B.); 2 Institut de Génétique et de Biologie Moléculaire et Cellulaire, UMR 7104 CNRS, U 964 INSERM, Université de Strasbourg, 67081 Strasbourg Cedex, France

**Keywords:** Murine Cytomegalovirus, Dicer, encephalitis, imaging

## Abstract

Among Herpesviruses, Human Cytomegalovirus (HCMV or HHV-5) represents a major threat during congenital or neonatal infections, which may lead to encephalitis with serious neurological consequences. However, as opposed to other less prevalent pathogens, the mechanisms and genetic susceptibility factors for CMV encephalitis are poorly understood. This lack of information considerably reduces the prognostic and/or therapeutic possibilities. To easily monitor the effects of genetic defects on brain dissemination following CMV infection we used a recently developed *in vivo* mouse model based on the neonatal inoculation of a MCMV genetically engineered to express Luciferase. Here, we further validate this protocol for live imaging, and demonstrate increased lethality associated with viral infection and encephalitis in mutant mice lacking Dicer activity. Our data indicate that miRNAs are important players in the control of MCMV pathogenesis and suggest that miRNA-based endothelial functions and integrity are crucial for CMV encephalitis.

## 1. Introduction

Human Cytomegalovirus (HCMV/HHV-5), a member of the β-herpesvirus family, is highly prevalent in the population and usually acquired during early life as an asymptomatic infection [1]. Like all herpesviruses, HCMV exhibits life-long persistence without viral replication in immunocompetent hosts. However, immunosuppression may lead to viral reactivation, as observed in transplant patients receiving drugs to prevent graft rejection [2]. In adults, HCMV has also been linked to glioblastomas [3]. Importantly, HCMV is also a prominent pathogen in newborns where the immune system is still immature [4,5,6]. Primary infection in the developing fetus or neonate can have severe consequences, such as microcephaly or cerebellar hypoplasia. Current estimates indicate that neonatal HCMV infection affects 0.5%–1% of all live births, of which 5%–10% will suffer from severe symptoms. As it stands, HCMV infection is the most common infectious cause of congenital birth defects and childhood disorders in developed countries. In addition, 10% of infected infants with subclinical viral infection will later develop sequellae leading to mental retardation, hearing loss, visual defects or seizure and epilepsy [7,8].

While a number of mutations responsible for the occurrence of neonatal Herpes Simplex 1 (HSV-1/HHV-1) encephalitis have been identified in humans [9], no genetic loci have so far been identified for HCMV, even though indirect evidence indicates that IL-12 and Type I interferons may be important players [10]. This discrepancy is likely related to the species specificity of HCMV—Indeed, as opposed to HSV-1, which can be inoculated to mice via different routes [11], non-human cells and organisms cannot support HCMV replication. Therefore, investigations on cytomegalovirus pathogenesis can only be performed in various animal models (mouse, rat, guinea pig, rhesus monkey) infected by their respective genuinely host-specific CMVs. In this regard, the mouse/MCMV interaction proved to be one of the best host—Pathogen models because of the significant similarities in genome size and organization, tissue tropism and regulation of gene expression between the murine and human viruses. These features provided considerable help in the identification of genes involved in CMV pathogenesis during infections in adults [12,13,14] reviewed in [15]. It must be noted that analysis of congenital CMV infection in mice appears to be more complex than analyzing the human form as the organization of the placental layers differs between the two species, with mother-to-fetus viral transmission being impaired in mice. Inducement of brain infection in mouse neonates, which in turn leads to hearing impairment, has been obtained upon direct injection of MCMV in the placenta on day 12.5 of gestation [16]. Most investigators, however, favor intraperitoneal injection of newborn (4–20 h-old) mice to provide systemic viral dissemination, which potentially leads to brain infection via the hematogenous route. This infection model proves to be more relevant than intracranial injection, as it provides important insights into MCMV encephalitis, especially in evidencing viral replication in neuronal and glial cells located in inflammatory foci which have been infiltrated with mononuclear cells, for example macrophages [17]. Previous work also reported altered morphogenesis of the cerebellum together with reduced granular neuron proliferation and migration as well as induction of multiple Interferon-stimulated genes, and further implicated that control of MCMV replication in the central nervous system requires CD8+T cells [18].

To gather more insight into genes and pathways involved in MCMV-dependent neonatal encephalitis we used our recently developed imaging technology based on the detection of a genetically-engineered MCMV expressing Luciferase (MCMV-Luc) to monitor and quantify viral replication *in vivo* [19]. Using this protocol, which allows for live imaging—Thus reducing the number of mice necessary for experimental infections [20]—We addressed the mechanisms of viral dissemination in the brain upon peritoneal infection of newborns. We focused these studies on the processing of microRNAs (miRNAs), *i.e.*, the 21–23 nucleotide non-coding RNAs which are—Among others—Major players in defense mechanisms [21]. Indeed, the recent discovery of virally-encoded miRNAs in the genome of large DNA viruses such as CMV [22,23,24] has considerably raised interest in these molecules in the context of virus–Host interactions. Therefore, we investigated the impact of a mutation in the *Dicer* gene, which is crucial for miRNA biogenesis [25], on the evolution of an intraperitoneal MCMV infection of newborn mice. In accordance with our previous observations in adults indicating that miRNAs are important players in antiviral defense [26], we here report increased mortality of Dicer-deficient neonates following viral inoculation. We also observed higher viral dissemination and replication in the brain of mutant pups compared to the wild-type, suggesting that miRNA biogenesis represents a previously unsuspected defense mechanism against Herpesvirus encephalitis.

## 2. Results

### 2.1. Impaired miRNA Biogenesis Induces Increased Lethality in MCMV-Infected Mouse Neonates

**Figure 1:**
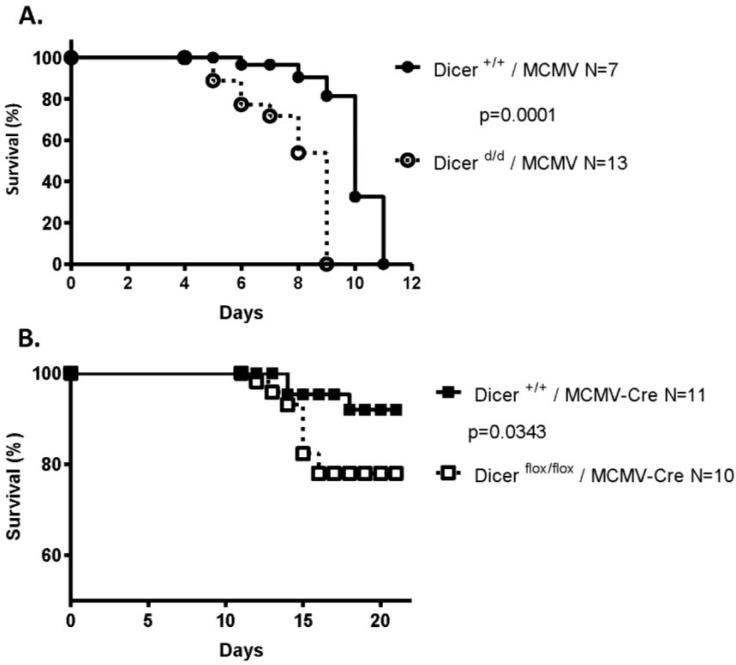
Increased lethality of Dicer-deficient newborns upon MCMV infection. (**A**). Control (Dicer ^+/+^, *N* = 7) and mutant (Dicer ^d/d^, *N* = 13) 4–8 h old neonates were infected by intraperitoneal injection of 50 p.f.u of MCMV (Smith strain, prepared *in vivo*). Survival of the pups was monitored every 12 h. (**B**). Dicer ^+/+^ (*N* = 11) and Dicer ^flox/flox^ (*N* = 10) were infected with 500 p.f.u of a recombinant MCMV-Cre (prepared by amplification in NIH 3T3 cells).

To evaluate the role of miRNAs on MCMV pathogenesis in newborns we first infected wild-type and *Dicer*-deficient (Dicer ^d/d^, [26,27]) 4–8 h-old pups with a highly pathogenic Smith strain of MCMV (*i.e.*, isolated from the salivary glands of infected Balb/c females) and monitored their survival. As shown in Figure 1A, postnatal development of wild-type mice was severely affected by the intraperitoneal (*i.p.*) injection of a small amount (50 p.f.u—Forming units) of MCMV, with all pups dying by day 11 post-inoculation. However, mortality was significantly (***, *p* = 0.0001) higher in mice carrying the Dicer ^d/d^ hypomorphic mutation. Virally-induced lethality is accompanied by decreased weight gain of both control and mutant pups compared to uninfected animals (Supplementary Figure S1A). To demonstrate that reduced *Dicer* expression is indeed responsible for this decreased viability upon viral infection in neonates we used a recombinant virus (MCMV-Cre), which allows for the expression of the Cre recombinase during the viral cycle. Injecting 4–8 h-old pups with this virus led to limited lethality in control wild-type mice (Dicer ^+/+^), most likely reflecting a reduced pathogenicity of the MCMV-Cre amplified in cultured cells. However, infection of Dicer ^flox/flox^ newborns led to significantly (*p* = 0.0343) increased lethality (Figure 1B) which, interestingly, is also accompanied by lower weight gain in the infected animals (Supplementary Figure S1B). The late onset of lethality in Dicer-floxed mice (starting at day 15) probably reflects the delay in the reduction of *Dicer* expression, as this requires gene excision and significant decrease in transcript and protein abundance. These experiments indicate that normal Dicer-dependent miRNA biogenesis is a crucial feature driving MCMV resistance. Furthermore, increased lethality of infected Dicer ^flox/flox^ neonates suggests that the mechanism is cell-autonomous and restricted to MCMV infected cells as it cannot be compensated by normal *Dicer* expression in non-infected cells.

### 2.2. Increased Viral Replication in Dicer-Deficient Newborns

**Figure 2:**
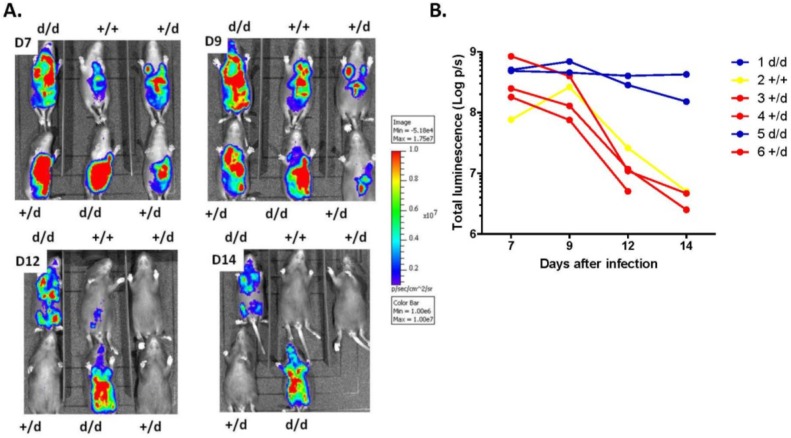
*In vivo* imaging reveals increased MCMV replication in infected Dicer-deficient neonates. (**A**). Snapshot images showing the luminescence emitted by MCMV-Luc-infected animals 7, 9, 12 and 14 days after infection. The genotype of the animals is indicated. (**B**). time-course analysis of luminescence (expressed in Log photons/sec or p/s) quantified for each animal presented in A. Wild-type control (Dicer ^+/+^, animal n° 2 in yellow), heterozygotes (Dicer ^+/d^, animals 3, 4 and 6 in red) and mutants (Dicer ^d/d^, animals 1 and 5 in blue).

To quantify viral replication *in vivo* we used a Luciferase-expressing MCMV (MCMV-Luc). 4–8 h-old wild-type (Dicer ^+/+^) and mutant (Dicer ^d/d^) neonates were inoculated *i.p.* with 500 p.f.u of this weakly pathogenic virus, after which viral amplification was monitored by way of Luciferin injection and subsequent quantification of light emission. *In vivo* imaging and data collection were performed on anesthetized pups at days 7, 9, 12, and 14 as described [19]. As seen in Figure 2A, while all pups of various *Dicer* genotypes (+/+; d/+ or d/d) exhibit similar Luciferase expression at day 4, imaging of the same animals at later time points reveals regular decrease of light emission in those carrying wild-type and heterozygous *Dicer* alleles. One Dicer ^+/d^ neonate died likely as a result of anesthesia. Remarkably however, the two Dicer ^d/d^ homozygous mutants identified in the litter used in this experiment exhibited sustained viral replication until day 14. A semi-quantitative representation of the light emitted by each animal is shown in Figure 2B, which clearly illustrates continuous viral gene expression in Dicer ^d/d^ neonates from day 7 to day 14. This indicates that while efficient viral clearance is observed in control animals, such mechanisms are lacking in pups with low *Dicer* expression. Similar data were obtained in 2 additional independent experiments in which the neonates were infected 12 h (Supplementary Figure S2A) or 24 h after birth (Supplementary Figure S2B). A compilation of the luminescence quantified for the different animals in these 3 experiments confirms increased viral replication in Dicer ^d/d^ neonates (Supplementary Figure S2C).

### 2.3. Preferential Viral Dissemination in the Brain of Dicer-Deficient Newborns

**Figure 3:**
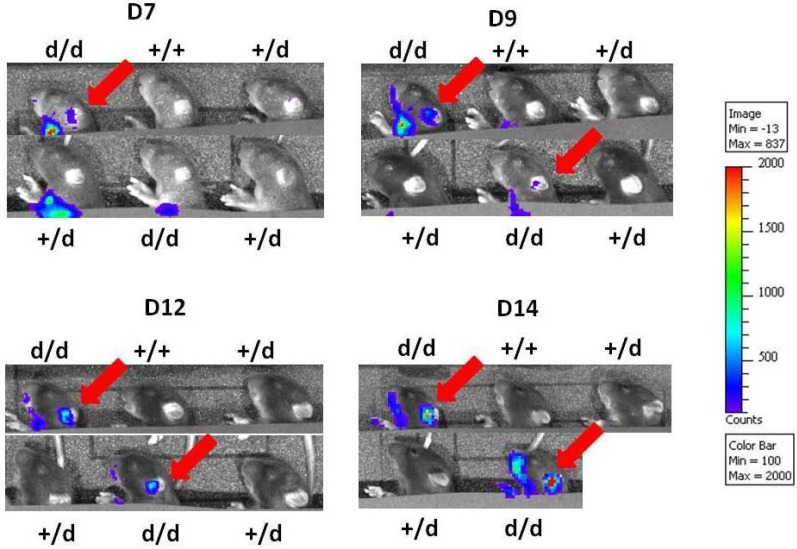
Preferential viral dissemination in the head of Dicer ^d/d^ mutants following MCMV neonatal infection. The abdomen of the animals was covered with dark cardboard to hide luminescence emitted from the lungs, spleen, liver or kidneys in order to increase the exposure time and to detect photons originating from the head region only. Data were obtained at days 7, 9, 12, and 14 following MCMV-Luc injection. Genotypes are indicated.

**Figure 4:**
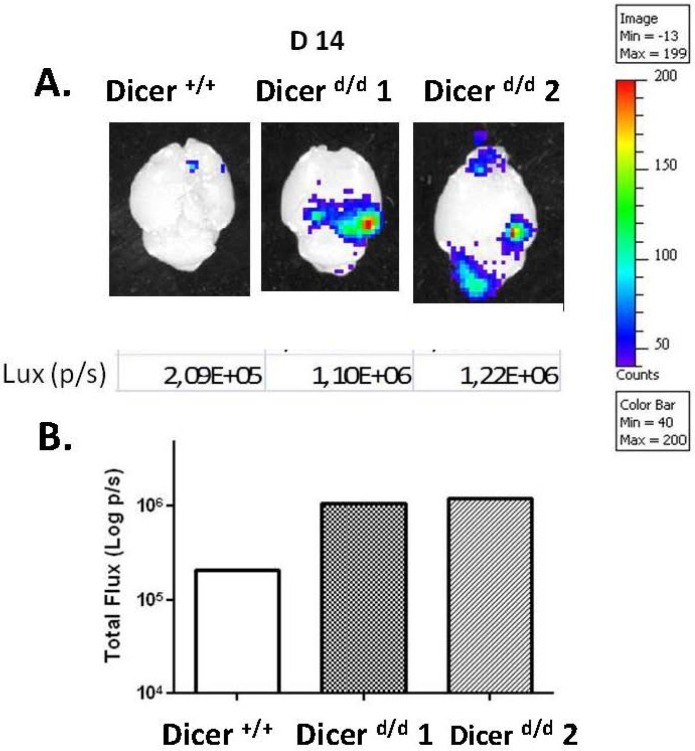
Increased brain infection in Dicer ^d/d^ mutants. (**A**). Snapshot image illustrating luminescence from the dissected brains harvested in a representative control neonate (Dicer ^+/+^) and two Dicer ^d/d^ mutants. (**B**). Luminescence (expressed in photons/sec or p/s) was quantified and plotted for each brain.

Live imaging of MCMV replication with high levels in large organs such as the lungs, spleen or liver may mask more discrete activity domains. We therefore masked the luminescence originating from the abdomen (using thick, dark cardboard) in order to detect a potential luminescent signal from the head alone, allowing the monitoring of possible viral replication in the brain. Increasing the length of the detection period enabled us to detect photons emitted in a discrete area at the ear level (Figure 3, red arrow). Interestingly, such a signal was visible only in Dicer ^d/d^ animals. To identify whether the brain is the source of this luminescence we euthanized the animals and recorded the light emitted from the dissected brains. As shown in Figure 4, strong signals are clearly visible in brains from Dicer ^d/d^ mutants, whereas a control brain shows negligible light emission. Quantification of these signals confirmed a marked (one Log) difference in signal intensity. Comparable results were obtained upon qPCR quantification of the viral genome (following the procedure described in [26]) in dissected brains harvested from a pool of MCMV-infected neonates (representing different experiments) at day 12. As shown in Suppl. Figure 3, increased (although not significant) viral replication can be observed in Dicer ^d/d^ mice. Surprisingly, one control neonate exhibited high viral titer in the brain, which can be the consequence of fortuitous hemorrhage during virus intraperitoneal injection and subsequent dissemination. Furthermore, the dissected brains were also used in RT-qPCR quantification (data not shown) to determine that *Dicer* expression is significantly reduced in mutants, which is similarly observed in other tissues [26,27].

**Figure 5:**
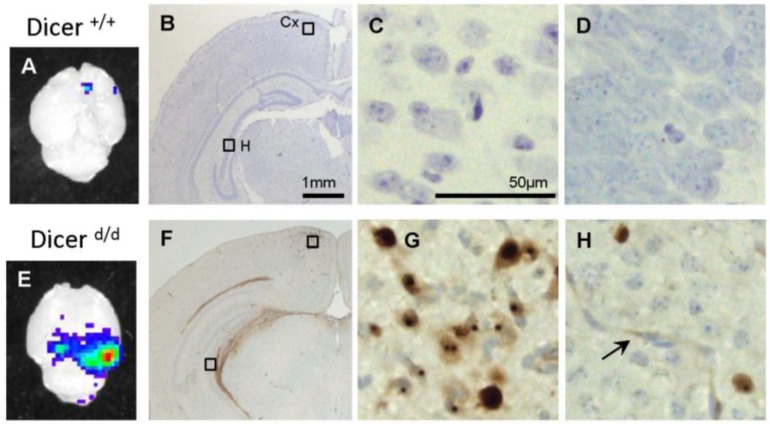
Immunohistochemistry reveal specific localization of MCMV in the brain of infected Dicer ^d/d^ neonates. (**A**) and (**E**). Snapshot images showing luminescence emitted by a control (Dicer ^+/+^) and a mutant (Dicer ^d/d^) brains respectively. Examples of immunohistochemical detection of E1 protein in Dicer ^+/+^ (**B**–**D**) and Dicer ^d/d^ brains (**F**–**H**) include macroscopic images (**B**), (**F**) and magnifications of selected regions of retrosplinal cortex (Cx; (**C**), (**G**)) and hippocampus (H; (**D**), (**H**)) at Bregma–3.08 mm. Immunolocalization of MCMV using the CROMA 103 primary antibody and peroxidase-labeled secondary antibody was depicted in brown and cresyl violet neuronal detection in blue. Epithelial staining in blood vessels is indicated by the arrow.

**Figure 6:**
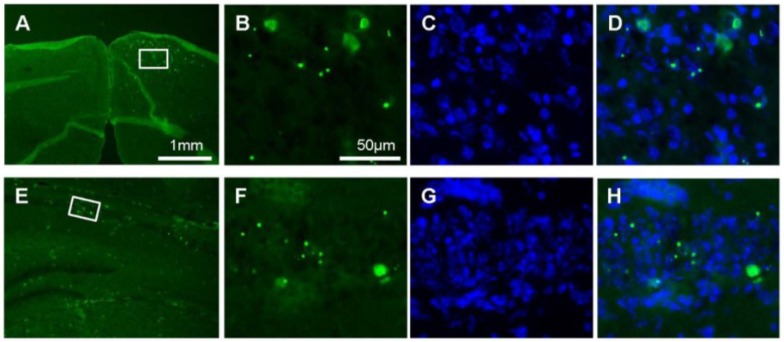
Nuclear detection of E1 protein in Dicer ^d/d^ mouse brain. Examples of immunofluorescent detection of E1 protein in the cortex ((**A**)–(**D**); Bregma—3.08mm) and hippocampus ((**E**)–(**H**); Bregma—2.3 mm) of Dicer ^d/d^ brain using CROMA 103 antibody. Magnifications of boxed regions illustrate E1 expression (**B**), (**F**), DAPI staining of the nuclear DNA (**C**), (**G**) and their colocalisation (**D**), (**H**).

To unambiguously demonstrate the presence of the virus in the brain and to locate its replication sites we performed Immunohistochemistry (IHC) staining on frozen brain sections from MCMV-inoculated Dicer ^+/+^ and Dicer ^d/d^ neonates. By using an antibody directed against the E1 protein (CROMA 103 Ab) we evidenced MCMV infection in the posterior brain region (bregma-3.08) in Dicer ^d/d^ mutants, which corresponds to the luminescent sites (Figure 5A,E). No staining was observed in the corresponding area of control brains (*i.e.*, from infected wild-type mice). Interestingly, mutant brains stained strongly not only in foci as illustrated for the retrosplenial cortex (compare Figure 5B–F and C–G), but also in the alveus of the hippocampus or in individual, dispersed cells in the granular layers of the hippocampus (compare Figure 5B–F and D–H). Although Nissl often co-localized with E1 protein in our experiment (Figure 5G,H), in good agreement with a previous report [28] showing that neurons are the primary target of MCMV infection in the brain, we cannot exclude that other cell types were also infected. We particularly observed frequent staining in elongated, epithelial-like cells in blood vessels of Dicer ^d/d^ brains (arrow in Figure 5H). Further immunofluorescence analyses revealed that the MCMV E1 protein was localized in nuclei (as detected by DAPI staining) in both the cortex (Figure 6A–C) and the hippocampus (Figure 6D–F).

## 3. Discussion

Neonatal infections by Cytomegalovirus induce major defects in multiple organs potentially causing premature death. Among the most severe complications that can occur are those associated with neurological dysfunctions (e.g., deafness or mental retardation). Due to their high prevalence CMV-related diseases in children represent a heavy burden; however the molecular mechanisms involved in CMV neuropathogenesis are less investigated than those caused by other herpesviruses. Data obtained by genetic analyses in children suffering Herpes Simplex 1 (HSV1) encephalitis provided clues on the physiopathology associated with this viral infection, leading to the identification of several genes involved in innate antiviral responses. Among these genes, those encoding for Tlr3 [29] or Unc-93B [30] could also be involved in the defense against other Herpesviruses such as CMV, even though this assumption has not been formally demonstrated. Additionally, mutations in these genes are extremely rare in the population and account for only a small fraction of neonatal HSV-1 diseases of genetic origin. Therefore, we used a preclinical approach and developed an *in vivo* imaging tool to screen for genetic (or pharmacological) factors in mice which affect MCMV dissemination to the nervous system, with the aim of identifying novel genes and pathways involved in CMV-related neurological abnormalities. In this report we describe increased lethality following intraperitoneal injection of MCMV in newborn mice where miRNA biogenesis was altered as a consequence of a hypomorphic mutation of the *Dicer* gene. Interestingly, we also observed a higher viral gene expression (as revealed by luciferase detection in the case of MCMV-Luc or E1 protein upon IHC experiments), reflecting [31] augmented virus titers in the brain of Dicer-deficient neonates. A precise localization of viral replication sites by IHC staining confirmed the viral infection of neurons localized in different cortical regions, including the entorhinal cortex and the hippocampus—Two areas tightly associated with cognitive functions [32]. While our work cannot directly link the increased death rate of the animals to the presence of MCMV in their brains we do however provide strong evidence for a major role of miRNAs in resistance to the infection. miRNAs are now considered as major regulators of gene expression and their role in antiviral immunity has been assessed in mammals [33,34,35], with our own work providing important insights into their role in the innate defense against viruses in adult mice [26,27]. However, the role of miRNAs in Herpesvirus infections is by nature difficult to investigate as they function both in the host immune defense as well as possible pathogenic factors encoded by the viral genomes. Therefore, studying MCMV infection in Dicer-deficient neonates, which are characterized by the relative immaturity of their immune system [18], provides for an interesting opportunity to understand the relative importance of both host and virally-encoded immune miRNAs. It can be concluded from our data that their role as pathogenic factors (whose decrease would be expected to lower MCMV aggressiveness) is dominated by that of host miRNAs (which likely exert protective functions). Several hypotheses can be drawn to account for the increased susceptibility of Dicer ^d/d^ neonates to MCMV infection and the virus’s preferential brain localization: 

(i) *A global innate immune alteration could be the consequence of low miRNA biogenesis, thereby provoking uncontrolled viral replication*. During these conditions, high viral load in the brain would simply reflect increased viral titers in organs such as the lungs, the kidneys or the spleen. In other words, high viral loads in the periphery would automatically drive a CNS infection. We do not favor this possibility because, as seen in Figure 2A, mouse number 2 (Dicer ^+/+^) exhibits very high luciferase levels in the abdomen (similar to those observed for Dicer ^d/d^ animals) and yet brain infection was not observed. Alternatively, we support a model in which.

*(ii) miRNA expressed by endothelial cells play a major role in the process of viral dissemination in the brain.* This is supported by the fact that MCMV replicates in these cells, contributing to viral dissemination [36]. Furthermore, miRNAs are important actors of endothelial cell functions [37]. Taking into account these observations, we speculate that endothelium perturbations in the blood-brain barrier represent a major issue, leading to MCMV leakage into the brain of Dicer ^d/d^ neonates. Because endothelium dysfunction can be either intrinsic to the Dicer ^d/d^ mutants or a consequence of MCMV infection future experiments aiming at exploring the integrity of the blood-brain barrier will be of high interest.

## 4. Materials and Methods

### 4.1. Mice and Ethics Statement

Animals were maintained under pathogen-free conditions in the animal care facility of the Institut d’Immunologie et d’Hématologie. Handling of mice and experimental procedures were conducted in accordance with the French Law for the Protection of Laboratory Animals. The procedure was approved by the service véterinaire de la Préfecture du Bas-Rhin (France) under the authorization number A-67-345. Dicer^d/d^ mice were described in [26,27]. Dicer ^flox/flox^ animals were described in [38].

### 4.2. Viruses

The MCMV Smith strain was amplified *in vivo* by three consecutive propagations in 3 week-old BALB/c females infected with 1 × 10^4^ plaque-forming units (p.f.u). Two weeks after intraperitoneal injection, salivary glands were harvested and homogenized in DMEM. Viral titers were quantified by plaque assay as previously described [39]. MCMV-Luc and MCMV-Cre (both provided by Lars Dölken, University of Cambridge, Cambridge, UK) were amplified in cultured NIH 3T3 cells and quantified by plaque assay in the same cells as described [39]. The insertion site of the Luc and Cre genes is reported in [40].

### 4.3. In Vivo Imaging

Infection of neonates and data acquisition of luminescence on an IVIS-50 (Caliper) were carried out as described [19]. Snapshot images and luminescence quantification (expressed in photons/sec—p/s) was performed with the dedicated software Living Image.

### 4.4. Immunohistochemistry Staining

Brains dissected from 14 days-old neonates were fixed overnight at +4 °C in 4% paraformaldehyde (PFA), washed in PBS and dehydrated by consecutive incubations in 70%, 95% and absolute ethanol followed by histosol. 7 µm thick coronal sections were prepared from paraffin embedded brains and collected on Superfrost Plus^®^ slides. For immunohistochemistry paraffin was removed and sections rehydrated in a series of baths including histosol followed by absolute, 95%, 70%, and 50% ethanol solutions, with subsequent termination by PBS incubation. Epitope retrieval was carried out by heating in citrate buffer (0.01 M, pH 6) with a microwave oven for 10 min, while endogenous peroxidase was inactivated by incubation in H2O2 1% in PBS, with non-specific sites saturated by incubation in Fetal Calf Serum (FCS) 7%. Primary antibody (CROMA 103; diluted 1/100; gift from Stipan Jonjic, School of Medicine, University of Rijeka, Croatia) was used to detect viral E1 protein and the signal was revealed using an ABC kit (Vector Laboratories, AbCys SA, France) according to the manufacturer’s protocol. Immunofluorescence experiments were performed using secondary antibody coupled with Alexa488 and DAPI for detection of nuclear DNA. Cresyl violet was used in counterstaining.

### 4.5. Statistical Analysis

GraphPad Prism 5.04 was used to perform statistical analysis of survival curves using Log-rank (Mantel-Cox) Test. Mann-Whitney (non parametric) test was used to compare two unmatched groups.

## Supplementary Files

Supplementary File 1
