# Supplementary material for: Increased Viral Dissemination in the Brain and Lethality in MCMV-Infected, Dicer-Deficient Neonates
Source: Viruses. 2015 May 6;7(5):2308–20. doi: 10.3390/v7052308 (PMC4452907; doi:10.3390/v7052308)

Legends to supplementary figures.

Suppl. Fig. 1. Low weight gain of MCMV-infected neonates. A. Time course analysis of the weight (expressed as a % of that measured at day 0) of control (Dicer $^{+/+}$ plain line) or Dicer-deficient (Dicer $^{d/d}$ dotted line) neonates following MCMV injection. As controls, the weight of uninfected animals is also shown (in blue). B. Time course analysis of the weight (expressed as a % of that measured at day 0) of control (Dicer $^{+/+}$, plain line) or Dicer-floxed (Dicer $^{flox/flox}$, dotted line) neonates following MCMV-Cre injection.

Suppl. Fig. 2. Increased viral replication in Dicer d/d neonates. A-B. Time-course analysis of luminescence (expressed in Log photons/sec or p/s) quantified upon MCMV-Luc injection in 12 h old (panel A) or 24 h old (panel B) neonates. Wild-type control (Dicer $^{+/+}$) are depicted in yellow), heterozygotes (Dicer $^{+/d}$) in red and mutants (Dicer $^{d/}$d) in blue). **C.** Comparison of the luciferase quantified at day 14 in mice from 3 independent experiments. * $p<0.05$.

Suppl. Fig. 3. MCMV genome quantification by qPCR in the brain of neonates 12 days post-infection. Controls (Dicer $^{+/+}$) are depicted as blue triangles; Dicer-deficient (Dicer $^{d/d}$) as red dots.

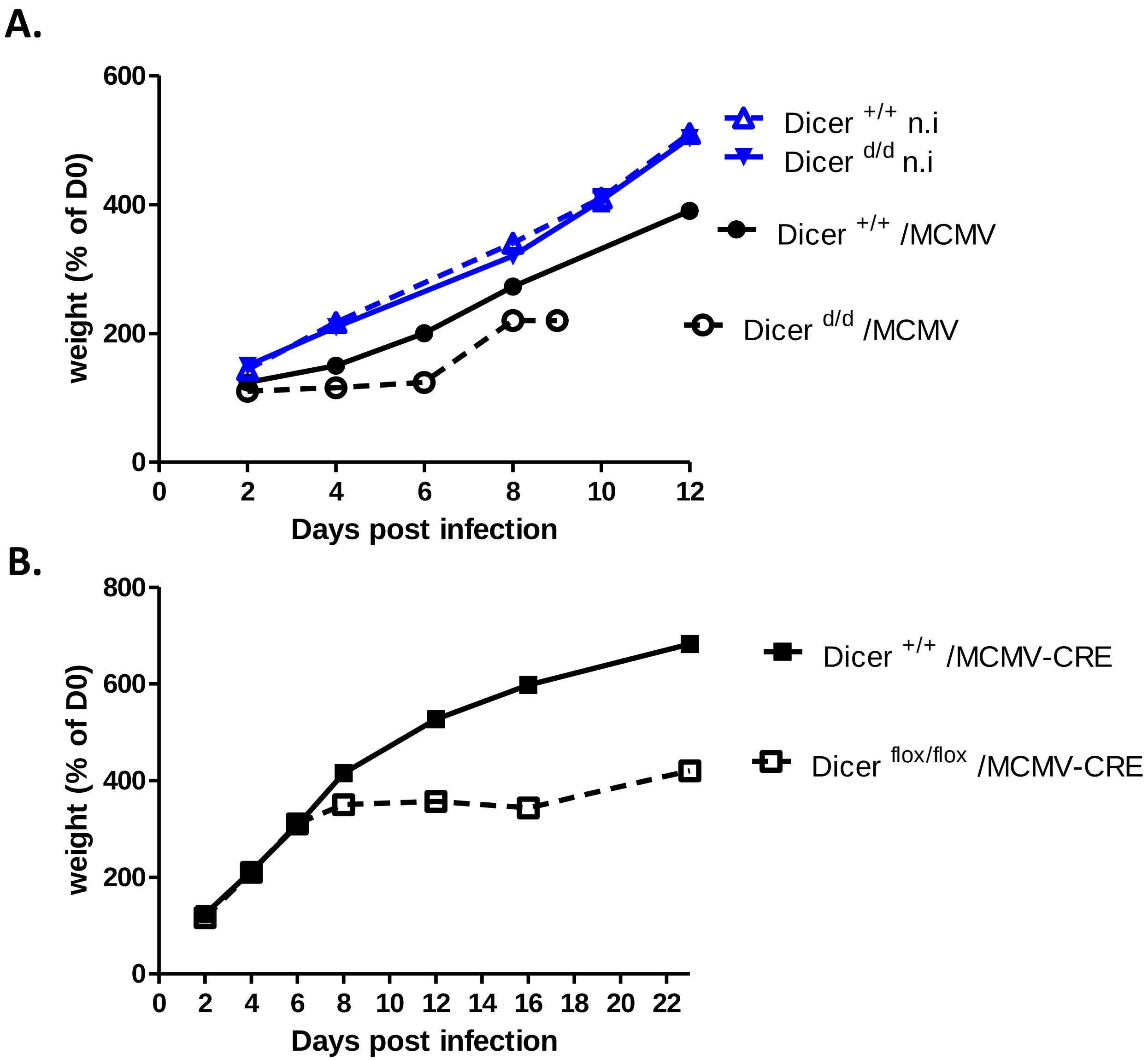
A.
weight (% of D0)
0
200
400
600
0
2
4
6
8
10
12
Days post infection
Dicer +/+ n.i
Dicer d/d n.i
Dicer +/+ /MCMV
Dicer d/d /MCMV
B.
weight (% of D0)
0
200
400
600
800
0
2
4
6
8
10
12
14
16
18
20
22
Days post infection
Dicer +/+ /MCMV-CRE
Dicer flox/flox /MCMV-CRE

Suppl. Fig. 1

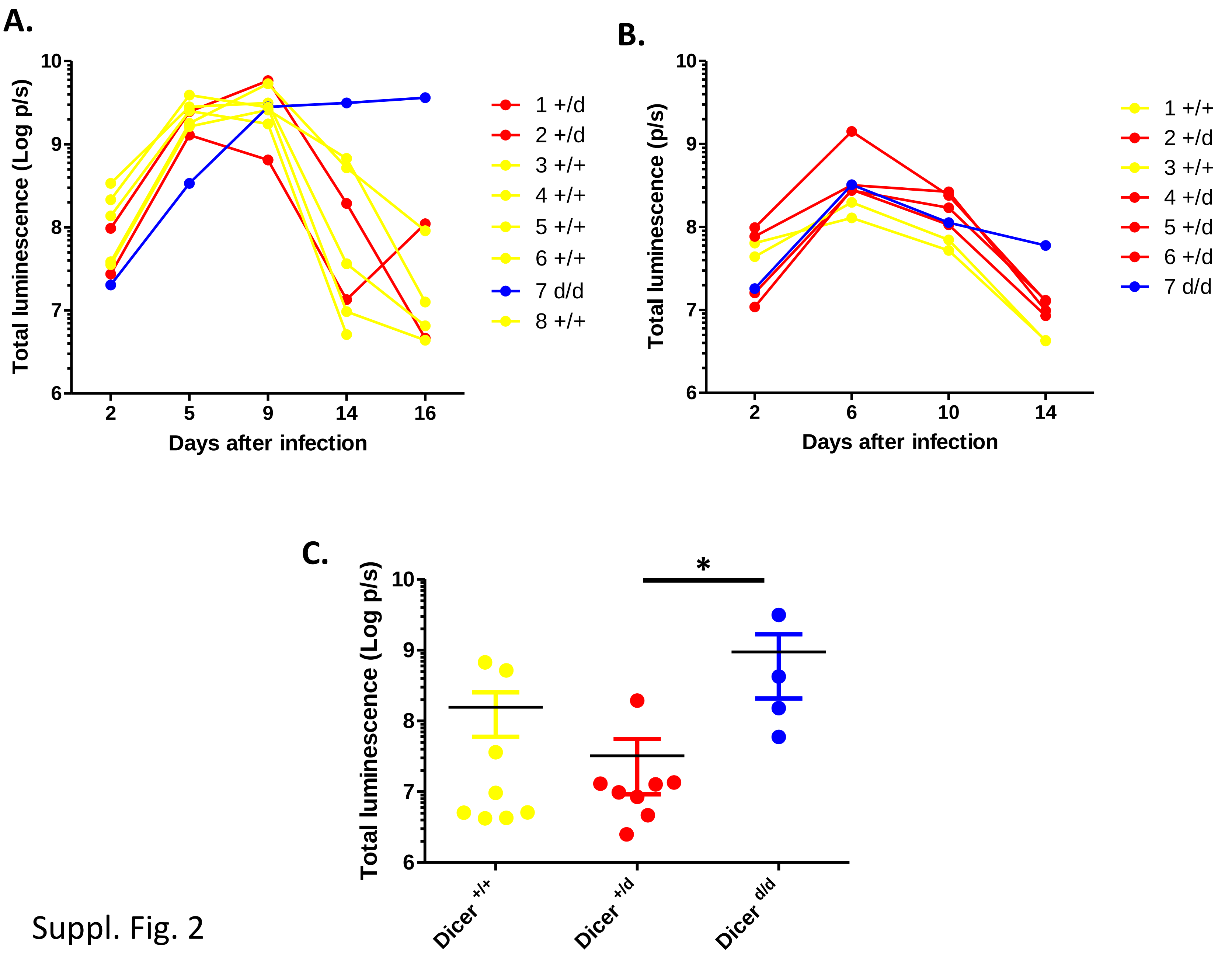
A.
Total luminescence (Log p/s)
Days after infection
1 +/d
2 +/d
3 +/+
4 +/+
5 +/+
6 +/+
7 d/d
8 +/+
B.
Total luminescence (p/s)
Days after infection
1 +/+
2 +/d
3 +/+
4 +/d
5 +/d
6 +/d
7 d/d
C.
Total luminescence (Log p/s)
*
Dicer +/+
Dicer +/d
Dicer d/d

Suppl. Fig. 2

Suppl. Fig. 3

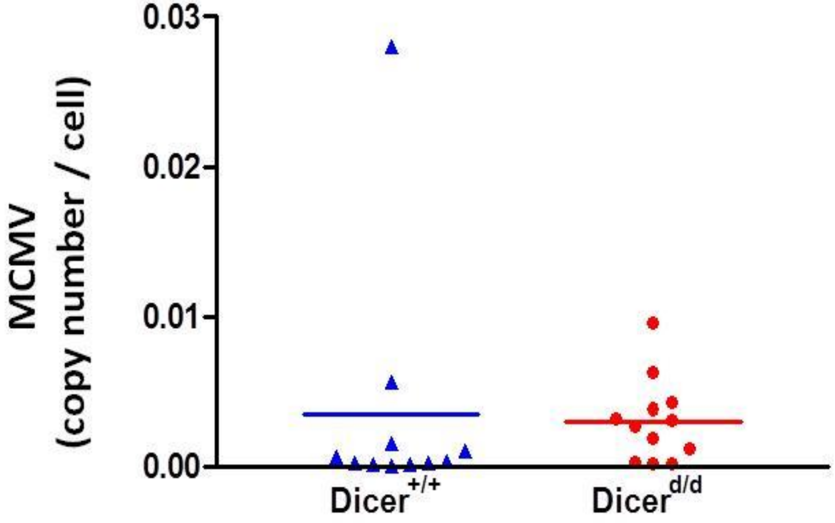

Supplement: Supplementary File 1 [file viruses-07-02308-s001.pdf]
